# Green Synthesis and Quality-by-Design Optimization of *Dacryodes edulis*-Derived Silver Nanoparticles with Broad-Spectrum Antiviral and Antimicrobial Activity

**DOI:** 10.3390/molecules31111821

**Published:** 2026-05-25

**Authors:** Jabulile H. Xulu, Vuyelwa J. Tembu, Sharon Moeno, Bienvenu Tsakem, Vuyisile S. Thibane, Bwalya A. Witika, Xavier Siwe Noundou

**Affiliations:** 1 Department of Pharmaceutical Sciences, School of Pharmacy, Sefako Makgatho Health Sciences University, Pretoria 0204, South Africa; jabuhappinessxulu@gmail.com (J.H.X.); btsakem23@gmail.com (B.T.); 2 Department of Chemistry, Faculty of Science, Tshwane University of Technology, Private Bag X680, Pretoria 0001, South Africa; tembuvj@tut.ac.za; 3 Department of Oral Biological Sciences, School of Oral Health Sciences, Faculty of Health Sciences, University of the Witwatersrand, Johannesburg 2193, South Africa; sharon.moeno@wits.ac.za; 4 Department of Biochemistry and Biotechnology, School of Science and Technology, Sefako Makgatho Health Sciences University, Pretoria 0204, South Africa; vuyisile.thibane@smu.ac.za

**Keywords:** *Dacryodes edulis*, quality-by-design optimisation, antiviral, antimicrobial, green synthesized silver nanoparticles, SARS-CoV-2, H1N1

## Abstract

The rising incidence of viral infections demands the creation of innovative, biocompatible antiviral drugs with broad-spectrum effectiveness. This study combines the green synthesis, optimization, and characterization of silver nanoparticles (AgNPs) utilizing *Dacryodes edulis* (*D. edulis*) extract, assessing their antiviral, and antimicrobial characteristics. AgNPs were synthesized through the bio-reduction of silver nitrate with *D. edulis* water extract as a reducing, capping and stabilizing agent. The synthesis was refined through a Design of Experiments methodology. The characterization techniques, UV-Vis, Fourier-transform infrared, transmission electron microscopy, and dynamic light scattering, validated the successful synthesis of AgNPs with an average size of 101.56 ± 28.22 nm (TEM) and 156 ± 0.81 nm (DLS), a polydispersity index of 0.34, and a zeta potential of −22 mV. High-resolution liquid chromatography–tandem mass spectrometry analysis identified some bioactive compounds which enhance the antimicrobial and antiviral properties of the samples. Enzyme kinetics experiments revealed substantial inhibitory efficacy against the SARS-CoV-2 papain-like protease (PL-pro), with AgNPs exhibiting a lower IC_50_ (0.271 ± 0.051 mg/mL) than the *D. edulis* extract (0.337 ± 0.043 mg/mL). The AgNPs exhibited MIC of 0.063 mg/mL for *E. coli*, 0.125 mg/mL for *S. aureus* and 0.08 mg/mL for *S. pyrogens*. The corresponding MBC values were 0.125 mg/mL, 0.25 mg/mL and 0.31 mg/mL, respectively. The fungal strains *C. glabrata* and *C. albicans* displayed MIC of 0.63 mg/mL and 0.31 mg/mL, respectively, and MBC values of 0.63 mg/mL and 0.31 mg/mL, respectively. This study underscores the potential of *D. edulis*-derived AgNPs as a cost-efficient, environmentally sustainable, and highly bioactive antibacterial and antiviral nanomaterial, facilitating the advancement of nanotechnology-based therapies for viral infections.

## 1. Introduction

It has been observed that over 88% of the global population still depends on medicinal plants for primary healthcare needs and the synthesis of various medicines [1,2]. While synthetic drugs can provide quick relief, they often come with numerous adverse side effects. Additionally, their high production cost makes them less accessible to a large portion of the world’s population [3,4]. Conversely, traditional medicines are typically regarded as safe, and easier for the body to metabolize [5]. Owing to people’s cultural and social beliefs, these medicines are readily available, affordable, and widely accepted [5,6,7]. The resurgence of infectious diseases, including viral infections, poses a serious threat to human life. Therefore, medicinal plants with wide-ranging antiviral properties have shown promise in fighting these dangerous diseases [8,9,10,11,12,13].

The common names for *Dacryodes edulis* are butter fruit tree, bush butter tree, African pear tree, African plum tree, safoutier (French), Eben tree (U.S.A.), and native pear (Africa) [14,15,16]. The *D. edulis* plant is native to Africa [17]. The stem bark of *D. edulis* is utilized as a decoction for treating tonsillitis, dysentery, anemia, and general oral hygiene. This decoction is also used as a gargle or mouthwash. Root and bark extracts have been employed in leprosy treatment, while a combination of boiled bark and leaves is used for malaria treatments. For wound healing, the bark is crushed and mixed with palm kernel oil [18,19,20]. The literature on *D. edulis* highlights the plant extract as an excellent source of natural therapeutic agents, providing a foundation for its application in modern medicine, i.e., nanotechnology. *D. edulis* displays extensive ethnomedicinal applications, particularly its traditional use in the management of microbial infections. Such practices highlight its therapeutic potential and provide a strong rationale for scientific investigations aimed at validating and characterizing its bioactive constituents. The bark of *D. edulis* was selected for this investigation as, based on the literature, it is the part that is traditionally used to treat viral infections [18,19,20]. Moreover, phytochemical analysis of the stem bark revealed a richness in polyphenolic compounds, renowned for their strong reducing and stabilizing properties. These bioactive constituents highlight the potential of the stem bark as a sustainable and eco-friendly source for green nanoparticle synthesis, functioning both as reducing agents for metal ion conversion and as capping agents to improve nanoparticle stability and functionality, further justifying our choice of investigating the stem bark of *D. edulis* in this study. To the best of our knowledge, this is the first report on the synthesis of AgNP-mediated *D. edulis* with applications in broad-spectrum antiviral and antimicrobial activities.

AgNPs have garnered significant attention due to the need for effective SARS-CoV-2 treatments. These nanoparticles (NPs) can be synthesized through physical, chemical, and biological methods. However, the chemicals used in their synthesis and stabilization are often toxic, expensive, and produce non-eco-friendly by-products [2,21,22,23]. Recently, biological methods have emerged as less toxic and environmentally friendly alternatives for the synthesis of AgNPs [2,24]. The green synthesis approach is particularly advantageous as it is cost-effective, eco-friendly, and can be easily scaled up for large-scale production without the need for high energy, pressure, temperature, or toxic chemicals [2,25,26]. More importantly, it is important to design, optimize and synthesize nanoparticles that meet some surface requirements in order to achieve the desired applications. To this end, the application of Quality-by-Design (QbD) optimization in nanoparticle synthesis is crucial for ensuring reproducibility, scalability, and consistent product quality. Unlike traditional trial-and-error methods, QbD employs statistical and systematic approaches such as Design of Experiments (DoE) to identify and control critical process parameters that influence nanoparticle size, stability, and bioactivity. This not only minimizes variability but also enhances efficiency, reduces resource consumption, and ensures that the final nanoparticles meet predefined performance criteria for effective biomedical applications.

Given the increasing reliance on medicinal plants for therapeutic applications and the need for sustainable nanoparticle synthesis, this study aimed to use a green synthesis approach to synthesize silver nanoparticles (AgNPs) using the stem bark extracts of *Dacryodes edulis* and evaluate their broad antiviral, antibacterial and antifungal activities. Throughout this work, the term “nanoparticle” is used to describe silver particles with dimensions of less than 1000 nm. This is consistent with broader definitions such as that of the British Standards Institution (BSI PAS 71:2011), which recognizes that materials larger 100 nm may still be classified as nanoscale when they exhibit nanoscale properties such as enhanced surface reactivity, surface plasmon resonance (SPR), and size-dependent bioactivity [27].

In this study, *Staphylococcus aureus* (ATCC 25923), *Streptococcus pyogenes* (ATCC 8665), *Escherichia coli* (ATCC 25922), *Pseudomonas aeruginosa* (ATCC 27853), *Candida albicans* (ATCC 90028), and *Candida glabrata* (ATCC 2850) were used. These strains were selected for their importance in hospital settings and the serious risk they pose to immunocompromised patients, including those with cancer. By targeting clinically prevalent strains that infect highly vulnerable groups, this study aims to support the development of new antimicrobial agents to address urgent clinical needs.

## 2. Results and Discussion

### 2.1. Phytochemical Screenings of D. edulis Water Extract

A qualitative phytochemical screening analysis was performed on this water extract to investigate which phytochemical classes were present in the stem bark of *D. edulis*. Table 1 provides a summary of the phytochemical properties of *D. edulis* water extract.

A number of studies have been done on the *D. edulis* plant species and revealed a variation in its phytochemical contents, such as phenolics, terpenoids, alkaloids and flavanols [15,16,19,20,21,22,23,24,25,26,27,28,29]. The variation in phytochemical content could be attributed to at least three different factors, i.e., different extraction methods, plant parts studied, and environmental factors [15,27,30]. The absence of saponins and steroids in the water extract might be due to the compounds being abundant in other parts of the plant, such as seeds or leaves [29,31]. Environmental factors like soil type, climate, and harvest season can also influence the presence of these metabolites [32]. The absence of these class of compounds might also be attributed to the sensitivity of the detection methods used [16,19]. This may have limited the identification of these compounds if present in low concentrations [32].

### 2.2. High-Resolution Liquid Chromatography–Tandem Mass Spectrometry (HR-LC-MS/MS) Analysis of the D. edulis Water Extract

**Figure 1:**
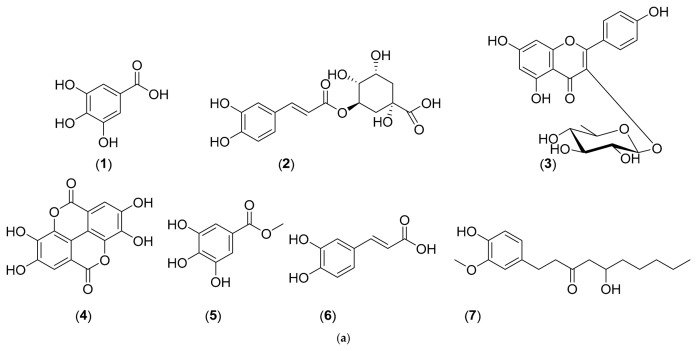

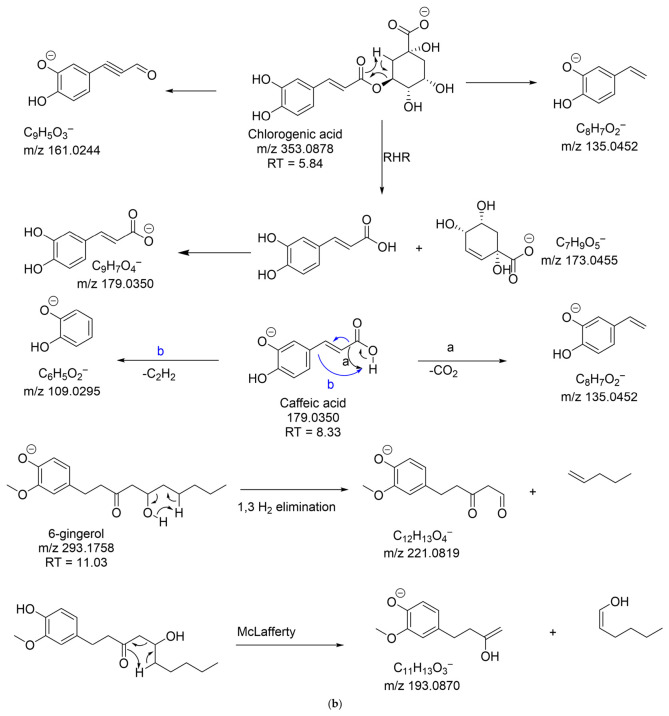
(**a**) Chemical structures of identified compounds from *D. edulis*; (**b**) the proposed MS/MS fragmentation patterns of theidentified compounds.

The total ion chromatograms (TICs) of the *D. edulis* water extract were obtained through electrospray ionization in negative mode (ESI^−^) and positive mode (ESI^+^). HR-LC-MS/MS analysis is one of the accurate techniques for precisely elucidating the chemical constituents of *D. edulis* water extract as it can analyze and separate compounds with higher sensitivity than other methods [33,34]. The compounds were successfully identified per their molecular ions (Table 2, Figure 1a). The total ion chromatogram is found in the Supplementary Materials (Figure S1).

The diverse phenolic compounds found in the *D. edulis* water extract indicate the broad-spectrum activity of the stem bark [35,36]. The presence of phenolic compounds in an extract is advantageous as phenols provide a natural source of diverse antimicrobial and antiviral activity, potentially leading to the development of new, wide-ranging therapeutic agents [37,38,39,40].

Carbon dioxide (CO_2_) is lost in the fragmentation pattern of ellagic acid, leading to fragment ions at *m*/*z* 259 and 217 [41]. A similar mechanism is observed with gallic acid and caffeic acid to produce, respectively, fragment ions at *m*/*z* 125 and 109. The fragmentation patterns of flavonoids follow the Retro-Diels Alder (RDA) mechanism to afford several possible ions [42]. The rearrangement of chlorogenic acid via retro-heteroatom reaction (RHR) led to the fragment ion at *m*/*z* 173. The McLafferty rearrangement in 6-gingerol provided the fragment ions at *m*/*z* 193, while the fragment at *m*/*z* 221 is obtained via 1,3 dihydrogen elimination [43]. The proposed fragmentation patterns of the elucidated MS^2^ compounds are illustrated in Figure 1b. Additional information on other possible fragments ions is found in the Supplementary Materials (Figure S2).

**Table 2:** Quadruple time-of-flight (QTOF) LC-MS/MS analysis of the detected metabolites from the *D. edulis* water extract.

Compound Name	Average Rt (min)	[M–H]^−^ and/or M+HCOO^−^ (*m*/*z*)	Exact Mass and Molecular Formula	MS/MS Fragments	References
Gallic acid (**1**)	3.27	169.0116	169.0142 C_7_H_5_O_5_	109.0273; 125.02020	[26,40,44]
Chlorogenic acid (**2**)	5.84	353.0485	353.0878 C_16_H_18_O_9_	135.0416; 161.0213 173.0434; 179.0345	[45,46,47,48]
Quercetin 3-*O*-α-L-rhamnoside (**3**)	6.28	477.1021	477.1038 C_22_H_21_O_12_	125.0215 389.0889	[49]
Ellagic acid (**4**)	7.39	300.9973	300.0999 C_14_H_5_O_8_	259.0269; 217.0524	[50,51,52,53,54]
Methyl gallate (**5**)	7.89	183.0246	183.0299 C_8_H_7_O_5_	125.0230 139.0419	[49,55,56,57]
Caffeic acid (**6**)	8.33	179.0344	180.0350 C_9_H_7_O_4_	109.0284 135.0416	[40,45,58]
6-gingerol (**7**)	11.03	293.1742	293.1758 C_17_H_25_O_4_^−^	143.1063; 221.1522 123.0400; 151.0765 193.0894	[59]

Gallic acid: HR-ESI-MS, *m*/*z* 169.0181 [M–H]^−^ with a molecular formula of C_7_H_6_O_5_ (calculated for *m*/*z* 170.0215), was previously isolated from the raw seeds of *D. edulis* [44]. This compound has broad-spectrum antiviral activity, including effectiveness against herpes and influenza viruses, supporting its potential against SARS-CoV-2 [40,60,61].

Chlorogenic acid: HR-ESI-MS, *m*/*z* 353.0485 [M–H]^−^ with a molecular formula of C_16_H_18_O_9_ (calculated for *m*/*z* 354.0878). Chlorogenic acid is known for its strong antiviral activity, inhibiting viral replication and preventing SARS-CoV-2 entry by blocking the spike protein-ACE2 receptor interaction [62]. This compound was also reported to have low toxicity, which further enhances its therapeutic profile making it a suitable compound to exhibit synergy with other compounds present [45].

Ellagic acid: HR-ESI-MS, *m*/*z* 300.9973 [M–H]^−^ with a molecular formula of C_14_H_6_O_8_ (calculated for *m*/*z* 302.0063). This compound was previously identified from the whole ripened fruit of *D. edulis* [49]. Ellagic acid is a therapeutic compound known for its antioxidant, anticancer, and anti-rhinovirus properties [49,52,63].

Methyl gallate: HR-ESI-MS, *m*/*z* 183.0426 [M–H]^−^ with a molecular formula of C_8_H_8_O_5_ (calculated for *m*/*z* 184.0372). This compound was previously identified from the whole ripened fruit of *D. edulis* [49]. Methyl gallate has been reported to inhibit bacterial adhesion and invasion, reinforcing its role in the antimicrobial properties of the extract [64,65].

Caffeic acid: HR-ESI-MS, *m*/*z* 179.0306 [M–H]^−^ with a molecular formula of C_9_H_8_O_4_ (calculated for *m*/*z* 180.0423). Caffeic acid is a valuable compound for antiviral treatment due to its ability to inhibit viral replication by targeting key viral enzymes, such as integrase, which is crucial for viruses like HIV [40,45,60].

The combination of various compounds found in the *D. edulis* water extract work together to enhance overall bioactivity [66,67]. These bioactive compounds contain hydroxyl groups in their structures, which have reducing properties and can engage in hydrogen bonding and other interactions due to their polar nature [53].

### 2.3. Experimental Design and Optimized Parameters

The optimization of biosynthesized AgNPs was conducted using a central composite design (CCD) to evaluate the interaction of significant variables and maximize synthesis efficiency [23]. Multiple experimental runs with varying critical process parameters (CPPs) and critical material attributes (CMAs) were analyzed using Design Expert^®^ to determine their impact on key formulation attributes. Data analysis identified the most influential variables and their optimal values, improving the formulation’s critical quality attributes (CQAs) [68]. The final optimization focused on ensuring desirable zeta potential (ZP), average particle size (PS), and polydispersity index (PDI), essential for maintaining the quality and performance of the formulation of the AgNPs.

The coefficient of determination (R^2^) values reflect how much variability in the observed response values can be attributed to the experimental factors and their interactions [69]. The standard deviation, R^2^ value, and coefficient of variation for the different responses are summarized in Table 3.

The quality of the models was assessed using the correlation coefficient, R^2^, and standard deviation values. The closer the R^2^ value approaches 1 and the smaller the standard deviation, the more precise the response that the model predicts. An R^2^ value greater than 0.9 indicates a good relationship between the experimental and predicted responses [70].

### 2.4. Response Surface Models’ Particle Size (Y_1_)

The significance of the response surface models was assessed through Analysis of Variance (ANOVA), utilizing the F-value of the model and the associated probability (Prob > F value) to ascertain its significance level at a confidence level of 0.05 [69]. The model F value was 5.93, which indicates that the model is significant. In this model, the particle size (PS) was significantly influenced by the increase in the concentration of AgNO_3_. A summary of the ANOVA results for the response surface quadratic model for PS is displayed in Table 4.

**Figure 2:**
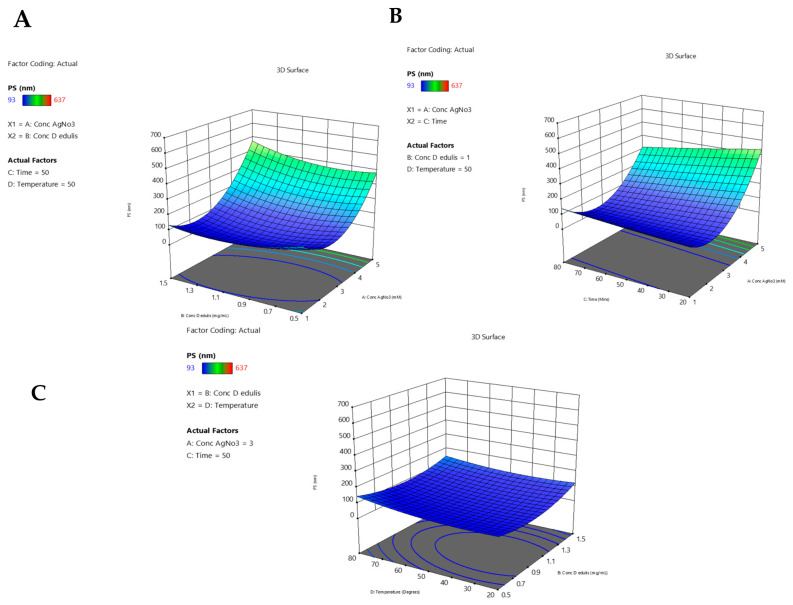
The 3D surface plot showing (**A**) the effect of the concentration of AgNO_3_ and *D. edulis* water extract, (**B**) effect of reaction time and concentration of AgNO_3_ and (**C**) effect of temperature and concentration of *D. edulis* water extract on particle size.

To investigate the effect of the independent variables on the PS of the AgNPs (dependent variable), three-dimensional (3D) surface plots are presented. In this regard, the graphs are presented in terms of variables that significantly affected the PS in the 3D surface plots in Figure 2.

The ANOVA data in Table 4 suggests that the most significant impact on the particle size was exerted by the concentration of the AgNO_3_ and the quadratic effect of the ionic concentration. This is further corroborated by the data presented in Figure 2 that illustrates a quadratic relationship between the concentration of AgNO_3_ and PS, where an increase in concentration results in an increase in PS. The concentration of *D. edulis* water extract does not significantly affect the PS. A reduction in both concentrations resulted in a particle size of less than 100 nm.

The reaction time exerted no significant influence on the particle size. The equation indicates a negative value for the parameter −18.21 C. This suggests that AgNP synthesis can be accomplished within a shorter duration without compromising particle quality. This finding is advantageous for process efficiency, reducing energy consumption and contributing to a more sustainable, cost-effective synthesis approach.

The interactions between each of the factors and the PS are summarized in Equation (1).

(1) $$Inverse\;Sqrt\;Y1=54.44+133.10\;A+7.46\;B-18.21\;C+19.25\;D+36.69\;AB-45.02\;AC+17.91\;AD\;\;\;\;\;\;\;\;\;\;\;\;\;\;\;\;\;\;\;\;\;\;\;\;\;\;+26.27\;BC-6.66\;BD+20.73\;CD+191.98\;A^{2}+51.29\;B^{2}+4.23\;C^{2}+22.47\;D^{2}$$

Mechanistically, in the presence of elevated Ag concentration, a substantial quantity of silver atoms is accessible for the growth of nucleated particles, facilitating the formation of bigger nanoparticles. The collision frequency of the generated particles markedly escalates with an increase in silver nitrate concentration. Consequently, the particles readily aggregate into bigger entities at elevated silver nitrate concentrations [71]. When the amount of phytochemical capping agents from *D. edulis* extract becomes insufficient relative to the growing particle surfaces, larger particles and broader size distributions are observed. This is consistent with earlier reports that the ratio of silver precursor to reducing/capping agents governs the extent of nucleation and growth, thereby controlling particle size and morphology [71,72].

### 2.5. Mechanistic Interpretation of Findings

The results indicate that both AgNO_3_ concentration and synthesis temperature markedly influence the size, surface charge, and stability of silver nanoparticles (AgNPs). At higher AgNO_3_ concentrations, the abundance of Ag^+^ ions accelerates reduction, favoring particle growth over nucleation. This produces larger nanoparticles with lower surface-to-volume ratios, limiting the adsorption of stabilizing phytochemicals and resulting in less negative zeta potentials, indicative of reduced colloidal stability. In contrast, moderate AgNO_3_ levels promote balanced nucleation and growth, generating smaller, well-capped particles with stronger negative surface charges. Similarly, elevated temperatures enhance reduction and phytochemical diffusion, initially supporting nucleation but ultimately leading to coalescence and phytochemical degradation, yielding larger, less stable nanoparticles. Moderate temperatures, however, enable controlled growth and effective capping, producing more stable AgNPs. Functionally, strongly negative zeta potentials improve electrostatic repulsion and colloidal stability, while larger, weakly charged nanoparticles may bind more strongly to microbial membranes but exhibit reduced systemic stability [73,74].

### 2.6. Response Surface Models for Polydispersity Index (Y_2_)

The ANOVA results in Table 5 show AgNO_3_ concentration as the only significant factor influencing PDI, with time and temperature being insignificant. The F-value is a measure of the model’s ability to explain variability in the response variable relative to unexplained variability. A higher F-value suggests that the model better explains the observed variation. However, in this case, the model’s *p*-value (0.1447) suggests that the factors examined may not collectively account for a significant portion of the response variability (Table 5).

The *p*-value (0.1447) of the model suggests that the factors examined may not collectively account for a significant portion of the response variability. The residual sum of squares (0.4609) and a mean square error of 0.0184 suggest that a considerable amount of variability remains unexplained by the model. This indicates the potential influence of other unaccounted factors or interactions not included in the model. The non-significance of the overall model (*p* = 0.1447) suggests that either higher-order terms, interactions, or additional factors may be necessary to improve the predictive ability of the model.

While the model in its entirety was not significant, the effect of AgNO_3_ was significant. As explained vide supra, when the amount of phytochemical capping agents from *D. edulis* extract becomes insufficient relative to the growing particle surfaces, broader size distributions are observed.

### 2.7. Response Surface Models for Zeta Potential (Y_3_)

The model for zeta potential (ZP) had an F-value of 3.39 which is significant. Statistical analysis confirmed that temperature and the combined effect of AgNO_3_ concentration and temperature significantly influenced ZP.

The ANOVA results for the response surface model for ZP are summarized in Table 6.

The data shows that the main contributing factor to the ZP was temperature. It is also observable that AgNO_3_ concentration and *D. edulis* concentration had *p*-values of 0.0726 and 0.0649, respectively, which are slightly above the 0.05 threshold, suggesting they may have a moderate influence but were not statistically significant in this model.

The interaction between AgNO_3_ concentration and temperature (AD) was highly significant (*p* = 0.0007, F = 16.35), suggesting a strong combined effect on ZP. This indicates that the impact of AgNO_3_ concentration on ZP is dependent on temperature, which could suggest that higher temperatures were required to drive the synthesis of NP and that directly affected the ZP. Other interactions (AC, BC, BD, and CD) and time did not show significant effects (*p* > 0.05). Higher concentration of AgNO_3_ provides more silver ions (Ag^+^) that can interact with functional groups on the NP surface providing more stability. Stable colloidal NPs with a ZP > |30| mV are less prone to aggregation or degradation over time due to the repulsive forces in the suspension [75]. This prolongs the shelf life of the synthesized NPs, reducing the need for frequent re-synthesis or use of polymers as stabilizers [76,77]. Stable NPs suspensions have good bioavailability and thus can be used for drug delivery applications [75]. The interactions between each of the factors and the ZP are summarized in Equation (2).

(2) ZP=3.65+14.37A+12.68B+5.12C−20.29D−16.23AB−5.43AC−39.01AD+4.44BC−16.60BD+13.79CD

**Figure 3:**
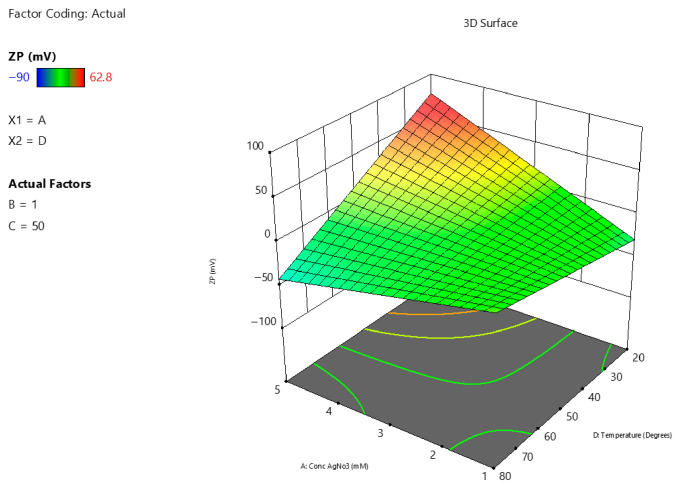
The 3D surface plot showing the effect of AgNO_3_ concentration and temperature on ZP.

The effect of AgNO_3_ concentration and temperature is represented in the 3D surface plots for ZP in Figure 3.

The coded regression (Equation (2)) demonstrates a significant negative main effect of synthesis temperature (D = −20.29) and an even more pronounced interaction between AgNO_3_ concentration and temperature (AD = −39.01) on ZP, suggesting that increased synthesis temperatures, especially at higher AgNO_3_ concentrations, result in more negative zeta potential values. Mechanistically, elevated temperatures expedite the reduction of Ag^+^ and the creation of nanoparticles while enhancing the adsorption and packing of deprotonated phenolics and tannins from the *D. edulis* extract, thus establishing a denser, anionic capping layer during synthesis. The AB (−16.23) and BD (−16.60) terms indicate that combining elevated precursor or extract concentrations with higher temperatures enhances negative surface charge, while the positive CD (+13.79) implies that at increased temperatures, extended reaction times can partially alleviate surface charge (e.g., through ligand rearrangement or ripening). Despite the positive main effects of A, B, and C at the design center, they are eclipsed by temperature-mediated interactions, as corroborated by the ANOVA analysis.

### 2.8. Response Surface Models for Surface Plasmon Resonance (Y_4_)

The model F-value was not significant, but the *p*-values indicated that the combined effect of concentration of AgNO_3_ and the concentration of *D. edulis* water extract was a significant combination. The ANOVA data for the response surface quadratic model for SPR is depicted in Table 7.

**Figure 4:**
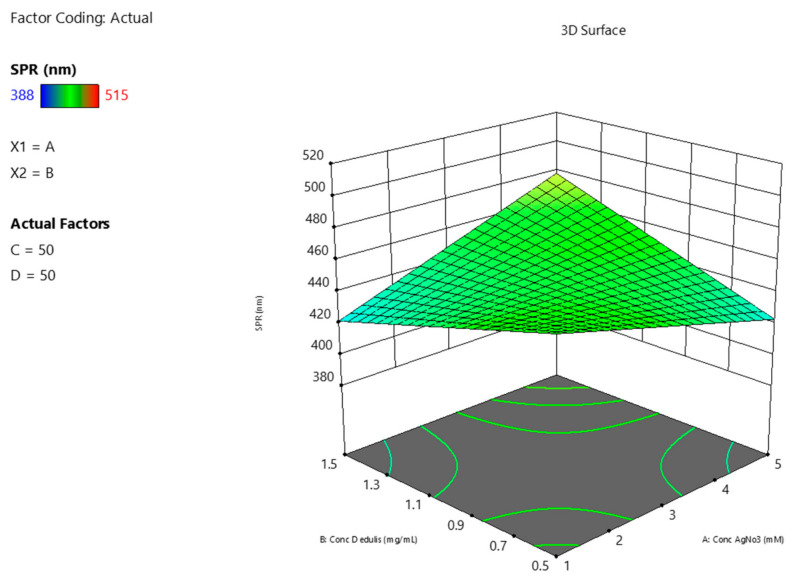
The 3D response surface plot depicting the impact of concentration of AgNO_3_ and *D. edulis* water extract on SPR.

The interaction of the concentrations of *D. edulis* and AgNO_3_ are visually represented in the 3D surface plots in Figure 4.

The concentration of AgNO_3_ and *D. edulis* extract significantly influenced SPR, indicating strong nanoparticle formation. SPR measurements confirmed successful synthesis and stabilization of biofunctionalized AgNPs.

### 2.9. Formulation Optimization

The numerical data was analyzed using the Design Expert^®^ software to determine which reaction conditions would yield the best responses in terms of SPR, PS, ZP, and PDI. The use of a numerical optimization methodology is a thorough and efficient method for a continuous optimization process [69,78]. The optimal conditions were found in the desirability zone, as indicated by the generated desirability of the model at 0.96. The synthesis parameters are crucial for synthesis of AgNPs that are non-agglomerated with a small shape and size. This is important for antiviral applications because they have better cellular uptake and interaction with viral proteins [79,80]. The parameters for the synthesis are summarized in Table 8.

From the optimized reaction conditions, AgNPs were then synthesized. The AgNP formulation was characterized for the nanoparticles’ optical properties, size distribution, surface functionalization, morphology, and crystallinity. These AgNPs were then used for the biological assays for the remainder of this study. The physiochemical activities of the AgNPs were compared alongside those of the *D. edulis* water extract. A summary of the CQA of the optimized AgNP formulation is provided in Table 9.

Despite the seemingly substantial percent differences at the optimum for certain responses (PS 13.86%, PDI 17.24%, ZP 26.67%), all noted deviations remain within one standard deviation of the model residuals (standardized residuals: PS 0.21, PDI 0.37, ZP 0.31, SPR 0.35), thereby residing comfortably within the 95% prediction intervals established by the ANOVA models. This signifies that the confirmation run is statistically aligned with model uncertainty. The comparatively greater percentage differences for PDI and ZP indicate the lower R^2^ of those submodels and the heightened sensitivity of these responses to minor modifications in synthesis and measurement (e.g., phytochemical batch composition, micro-mixing, and post-processing).

### 2.10. Characterization of the AgNPs

#### 2.10.1. Ultraviolet–Visible Spectroscopy Analysis

**Figure 5:**
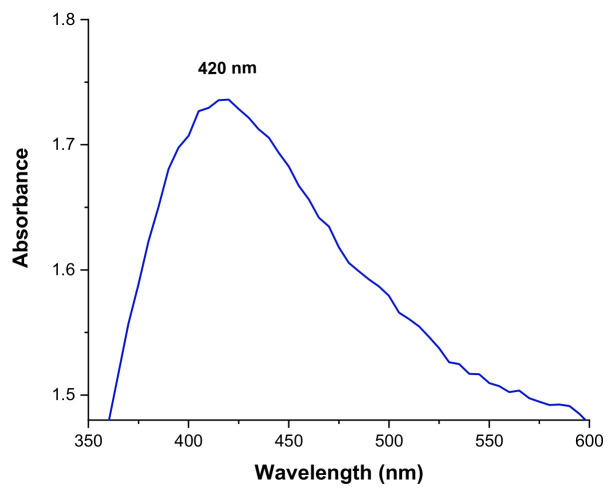
UV-vis spectra of the dispersion of optimized AgNPs.

The UV-vis spectra in Figure 5 shows defined surface plasmon resonance (SPR) bands centered at 420 nm, an indication of the characteristic peak of AgNPs.

The characteristic peak at 420 nm was indicative of the reduction of Ag^+^ to elemental silver (Ag^0^), indicating the formation of AgNPs. The different shapes of AgNPs have also been noted to directly affect the intensity of the absorbance [81]. Mie’s theory states that anisotropic particles can produce two or more SPR bands depending on their shape, whereas spherical metal NPs can only produce one SPR band [82]. This variation in SPR further highlights the impact of the shape of NPs on their optical properties.

#### 2.10.2. Particle Size, Distribution and Zeta Potential

**Figure 6:**
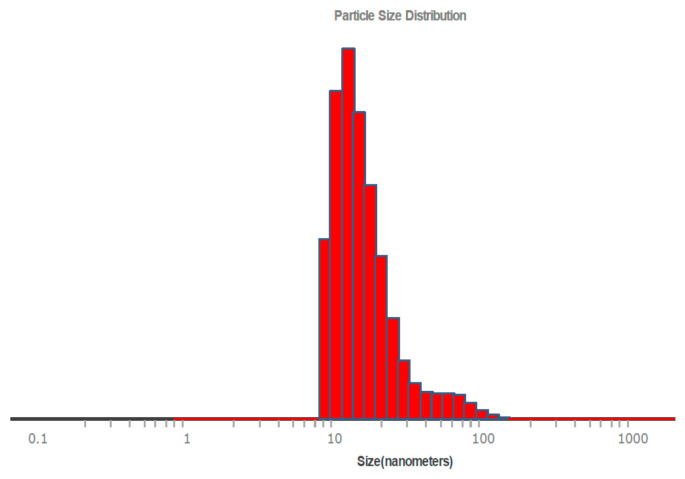
DLS histogram of the optimized AgNPs.

Dynamic light scattering (DLS) was used to characterize the particle size and particle size distribution (PSD) of biosynthesized AgNPs. The colloidal AgNPs were measured and expressed as average particle size distribution, as shown in Figure 6.

The mean intensity (MI) of the particles was found to be 156 ± 0.81 nm. AgNPs in this size range are commonly used for their biomedical applications because they can bind viral/protein enzymes to their surface [83,84,85,86]. While some of the biosynthesized AgNPs exceeded 100 nm in size, they are classified as nanoparticles as a consequence of their synthesis via a biogenic, bottom–up nanoscale process and the presence of characteristic nanoscale characteristics, including a defined SPR peak, enhanced antimicrobial and antiviral activity, and increased surface area-to-volume ratios.

The PDI value of 0.34 indicates that the size distribution is relatively narrow and monodisperse, which is beneficial in many applications as it suggests a more uniform population of NPs in terms of their size distribution in solution [87,88]. A zeta potential of −22 mV indicates moderate stability due to the phenomenon of particle electrostatic repulsion [22,58,89]. A negative zeta potential indicates that they are negatively charged, which creates repulsive forces between the NPs. This repulsion force acts against the attractive forces that may otherwise cause flocculation or particle aggregation [75,90]. This means that nanoparticle formulation is stable, but under some conditions might be at risk of aggregation.

Taken together, the PDI value, zeta potential, and defined SPR band provide a preliminary indication of colloidal stability of the optimized AgNP dispersion. However, no dedicated short-term stability assessment or release study in a physiologically relevant medium was performed in the present work. Accordingly, these findings should be interpreted as an initial physicochemical indication of stability rather than a full stability or biorelevant release evaluation, which remains important for future studies.

#### 2.10.3. Transmission Electron Microscopy

**Figure 7:**
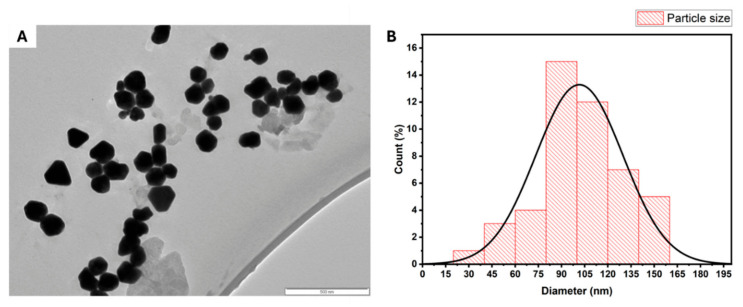
TEM micrographs of the optimized AgNPs (**A**) and the TEM-derived size distribution (**B**).

Image J^®^ software version 1.54e (National Institutes of Health, Bethesda, MD, USA) was used as an image-processing tool to analyze the bio-capped AgNPs. The morphology and size of AgNPs were analyzed by TEM, displaying polymorphic shapes and wide-size distribution, as shown in Figure 7.

TEM images showed that most of the biosynthesized AgNPs were spherical to quasi-spherical in shape, with a few triangular and rod-shaped identified. Spherical and quasi-spherical NPs offer high surface area, better colloidal stability, and efficient cellular uptake, making them ideal for broad-spectrum antiviral activity [91,92]. AgNPs with various sizes and shapes have been reported to display the highest antibacterial effect on different pathogens [76]. The NPs had sizes ranging from 20 to 160 nm. The AgNP formulation exhibits a mean particle size of 101.56 ± 28.22 nm, as shown in the size distribution histogram of the AgNPs.

#### 2.10.4. Scanning Electron Microscopy and Energy-Dispersive X-Ray Scanning Electron Microscopy

**Figure 8:**
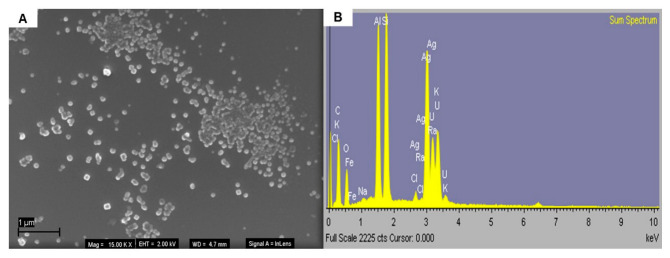
A visual depiction of SEM micrograph (**A**) and EDX spectrum (**B**) of the AgNPs.

SEM analysis was performed to visualize the surface morphology of the AgNPs, while EDX was used to assess the elemental composition of the AgNP formulations. The SEM micrograph and EDX spectrum are depicted in Figure 8.

During the SEM analysis, it was observed that the surface of the AgNPs was not smooth, and the NPs are partly agglomerated. The EDX detector on the microscope allowed for gathering of information pertaining to the elemental composition of the sample. The Ag peak was detected strongly around 3 keV, which is a common finding in literature where Ag is studied [93,94,95,96]. The EDX analysis detected a strong presence of silver, indicating that silver ions were successfully reduced to elemental silver during the nanoparticle synthesis [95,97].

#### 2.10.5. Fourier-Transform Infra-Red Spectroscopy

**Figure 9:**
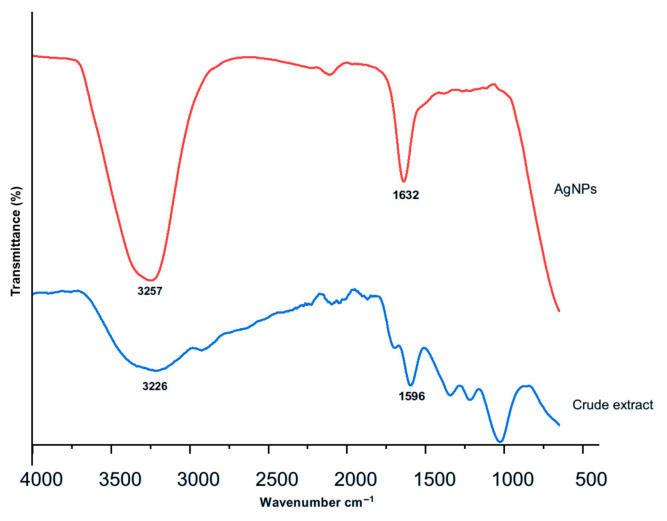
FTIR spectra of synthesized AgNPs and *D. edulis* water extract.

The functional groups responsible for reduction, stabilization, and capping agents were confirmed by FTIR analysis. The 500–4000 cm^−1^ range of the FTIR absorption spectra of green synthesized AgNPs and *D. edulis* water extract was examined and is presented in Figure 9.

The absorption band at 3226 cm^−1^ in the *D. edulis* water extract indicates the presence of hydroxyl (OH) groups, with its broadness suggesting multiple O–H bonds. The 1596 cm^−1^ band corresponds to the aromatic C=C stretch, consistent with previous reports. In the AgNPs spectra, a broad band around 3257 cm^−1^ is attributed to OH stretching of phenolic compounds or N–H stretching vibrations from amino groups in the *D. edulis* water extract. The 1632 cm^−1^ band is associated with carbonyl (C=O) stretching or N–H bending in primary amides. The displacement at 3257 cm^−1^ may indicate hydrogen bond breaking during the reduction of silver ions to AgNPs. The similarity in spectral patterns between the extract and the synthesized AgNPs suggests the involvement of phytochemicals from *D. edulis* water extract in the nanoparticle synthesis.

#### 2.10.6. Powder X-Ray Diffraction

**Figure 10:**
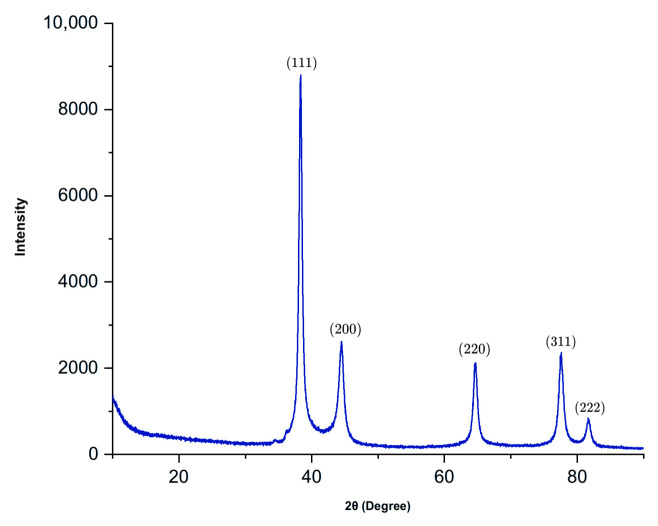
*p*XRD pattern of the AgNPs.

The crystalline structure, size and morphology of the synthesized AgNPs were assessed using *p*XRD analysis. The *p*XRD pattern for the AgNPs that were synthesized with *D. edulis* water extract is depicted in Figure 10.

The presence of certain diffraction peaks at specific 2θ values, such as 38.34°, 44.29°, 64.43°, 77.30°, and 81.58°, indicates that the AgNPs have a crystalline structure, as reported in literature [98,99,100,101]. In literature, crystalline AgNPs are reported to be less cytotoxic and possess potential anti-viral and anti-bacterial activity [102,103,104]. The identification of diffraction planes, or hkl values, provides information about how the atoms are arranged in the crystal lattice [21,105]. In this case, the observed peaks are associated with the (111), (200), (220), (311), and (222) planes commonly associated with silver [98,106]. The observed peaks follow Bragg’s law, which describes the constructive interference of X-rays scattered by crystal planes [21,107,108,109,110]. The estimated average crystalline size (D) of the optimized AgNPs was calculated using the Deybe–Scherrers equation. The average crystallite size was an average of 78.80 ± 0.95 nm (*n* = 5). The variation between the size of the AgNPs across different measurement techniques indicates that factors such as particle aggregation or dispersion conditions might affect the measurements [111]. This is important when considering these factors during characterization of nanoparticles.

### 2.11. Biological Assays

#### 2.11.1. Cell Viability Assay

**Figure 11:**
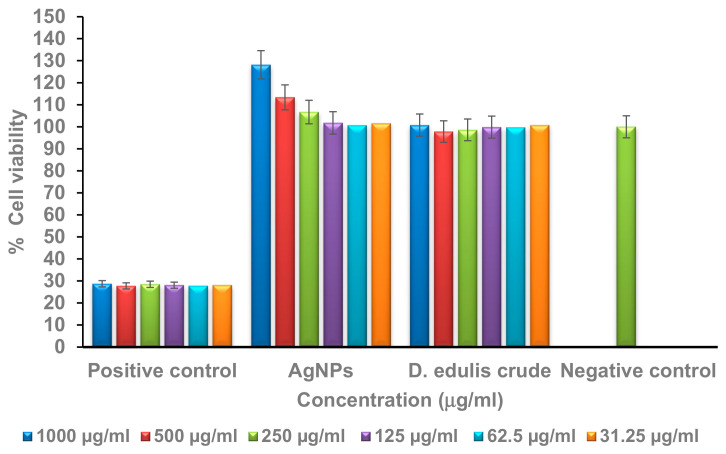
Cell viability assay of optimized AgNPs and *D. edulis* water extract on HEK 293 cells.

The MTT cytotoxicity assay was performed to assess the cell viability of cells treated with *D. edulis* water extract and AgNPs. The cell viability of the *D. edulis* water extract and AgNPs was assessed on the human embryonic kidney (HEK) 293 cell line. The graph of the cell viability is depicted in Figure 11.

The data depicted in Figure 9 suggests that the AgNPs used in this study are biocompatible, leading to increased uptake by cells, potentially causing the proliferation of the HEK-293 cell line. A decrease in *D. edulis* water extract concentrations did not affect cell viability in the concentration test, and no clear dose–response relationship was observed. This suggests that, under these conditions, the *D. edulis* water extract does not influence cell viability in a concentration-dependent manner. Overall, the results indicate that both the *D. edulis* water extract and AgNP formulation show no signs of toxicity to HEK cells. At concentrations of 1000 μg/mL and 500 μg/mL, AgNPs promote cell proliferation, possibly due to the surface modifications induced by the plant extract and its bioactive compounds.

#### 2.11.2. Papain-like Protease Enzyme Assay

The Papain-like protease enzyme assay monitors the enzymatic activity of PL-pro by measuring the amount of cleaved product that is generated [112,113]. This assay analyzed the inhibitory effects of the AgNPs and *D. edulis* water extract when incubated with the PL-pro and substrate over varied periods. The IC_50_ values are calculated and represented in terms of mean ± standard deviation (SD) and summarized in Table 10.

The IC_50_ for *D. edulis* water extract and its derived-AgNPs were significantly different when compared to each with values of 337.0 ± 23.00 mg/mL and 271.0 ± 15 mg/mL, respectively. The positive control expressed a potent antiviral activity with an IC_50_ value of 0.487 µg/mL. Both the *D. edulis* extract and AgNPs demonstrated inhibitory activity, with AgNPs showing greater potency in inhibiting enzymatic activity. The lower IC_50_ for AgNPs suggests a higher affinity for modulating the PL-pro enzyme compared to the *D. edulis* extract, likely due to the size and shape of the nanoparticles enhancing their attachment to the enzyme surface. The *D. edulis* extract is rich in bioactive compounds with therapeutic potential, and its low IC_50_ highlights its promise as a natural antiviral source. This suggests that the AgNPs have a higher affinity in modulating the PL-pro enzyme as compared to the *D. edulis* water extract [79,114]. The improved PL-pro inhibitory activity of the AgNPs relative to the crude extract may therefore be understood as arising not only from the intrinsic antiviral potential of the *D. edulis* phytochemicals, but also from nanoparticle-related effects such as increased surface area and favorable particle–enzyme interactions. In this context, the biofunctionalized AgNP surface may facilitate stronger interaction with the PL-pro enzyme, thereby enhancing inhibitory activity compared with the plant extract alone.

The results support the hypothesis that silver nanoparticles synthesized from *D. edulis* water extract possess anti-SARS-CoV-2 activity, aligning with growing interest in plant-derived compounds for viral infections.

#### 2.11.3. Neuraminidase Assay

The AgNPs and the *D. edulis* water extract were analyzed for antiviral efficacy against the H1N1 neuraminidase assay. The IC_50_ values are calculated and represented in terms of mean ± standard deviation (SD) and summarized in Table 11.

The absorbance values of both the AgNPs and *D. edulis* water extract indicated a concentration-dependent response. The commercially known positive control (Oseltamivir) expressed potent antiviral activity against neuraminidase with IC_50_ values of 0.1769 ± 0.04 µg/mL. The antiviral activities of both the AgNPs and *D. edulis* water extract were significantly lower than those of the positive control. However, the AgNPs expressed a significantly higher inhibitory effect on the neuraminidase activity when compared to *D. edulis* water extract with IC_50_ values of 18.40 ± 0.04 and 514.39 ± 86.37 µg/mL. The AgNPs exhibit inhibitory activity, which indicates increased enzyme inactivation through binding with the neuraminidase enzyme. On the other hand, *D. edulis* water extract exhibits notable inhibitory activity, which is indicative of the antiviral activity of the plant extract and its subsequent high therapeutic potential.

Similar to the PL-pro mechanistic outlook, the AgNPs relative to the extract alone may reflect improved enzyme inactivation through nanoparticle–enzyme interaction, together with the contribution of antiviral phytochemicals associated with the *D. edulis*-derived capping layer. Thus, the antiviral response observed in this study is likely a function of both the silver nanoparticle platform and the bioactive plant-derived surface chemistry.

#### 2.11.4. Antimicrobial Assay

The AgNPs and the *D. edulis* water extract were evaluated for their antimicrobial activity against four bacterial, and two fungal strains (*Staphylococcus aureus* (ATCC 25923), *Streptococcus pyogenes* (ATCC 8665), *Escherichia coli* (ATCC 25922), *Pseudomonas aeruginosa* (ATCC 27853), *Candida albicans* (ATCC 90028), *Candida glabrata* (ATCC 2850)). The results for the MIC and minimal bactericidal concentrations (MBCs) are summarized in Table 12.

The AgNPs exhibited MIC values of 0.063 mg/mL for *E. coli* and 0.125 mg/mL for *S. aureus*. The corresponding MBC values were 0.125 mg/mL and 0.25 mg/mL, respectively. These results indicate that the AgNPs not only inhibit bacterial growth but also exhibit bactericidal activity [115,116]. The fungal strains *C. glabrata* and *C. albicans* displayed identical MIC and MBC values of 0.63 mg/mL and 0.31 mg/mL, respectively. These findings suggest that the AgNPs possess fungistatic properties, effectively inhibiting fungal growth without completely eliminating the fungi [115,117].

The *D. edulis* water extract showed broad-spectrum antimicrobial efficacy against the bacterial and fungal strains. The different microorganisms displayed varied MIC and MBC values, indicating diverse susceptibility levels to the plant extract. The *E. coli*, *P. aeruginosa*, *S. pyogenes* and *C. albicans* strains appear more sensitive, with lower MIC values at 0.63 mg/mL for all strains, while *S. aureus* and *C. glabrata* have MIC values of 1.25 mg/mL for both strains. The MBC values for the *D. edulis* water extract are 2 folds or even 4 folds higher than the observed MIC values, which indicates that the *D. edulis* water extract is both bactericidal and fungicidal. The *D. edulis* water extract exhibits its broad-spectrum antimicrobial efficacy possibly through the vast combination of phytochemicals present in the crude [16,20,28,31,44,117,118]. Based on previous literature and the metabolites identified in the *D. edulis* extract, the observed antiviral and antimicrobial activities may plausibly be associated with phenolic constituents such as gallic acid, chlorogenic acid, caffeic acid, ellagic acid, and methyl gallate, which are widely reported to have anti-infective relevance. These compounds may contribute directly to biological activity and may also enhance nanoparticle functionality by participating in reduction, capping, and surface biofunctionalization during AgNP synthesis. In addition, 6-gingerol and the detected flavonoid glycoside may provide further supportive or synergistic contributions [119,120,121,122]. Accordingly, the biological effects observed in the present study are more likely due to the combined action of the AgNP platform and the associated phytochemical corona, rather than a single isolated constituent.

The broad-spectrum antimicrobial activity observed for the AgNPs is likely attributable to the combined effects of the bioactive silver nanoparticle surface and the phytochemical constituents derived from *D. edulis*, which may act synergistically. The relatively small particle size, moderate colloidal stability, and phytochemical capping may enhance interaction with microbial targets and contribute to the improved activity of the AgNPs compared with the crude extract alone.

Ciprofloxacin was used since it is a broad-spectrum fluoroquinolone antibiotic used to treat various bacterial infections, while Amphotericin B, a polyene antifungal, was selected due to its application in treating fungal infections. The starting concentration used for each compound and control drugs was 2.5 mg/mL, and the negative control used was a broth, either Mueller Hinton broth (MHB) for bacteria or Sabouraud dextrose agar (SAB) for fungi. The activity of Ciprofloxacin was tested against the bacterial strains. For *S. aeruginosa*, additional serial dilutions were prepared until MIC and MBC values were determined at the lowest tested concentration of 0.005 mg/mL. *Escherichia coli*, *Staphylococcus aeruginosa*, and *Streptococcus pyogenes* exhibited inhibitory concentrations of 0.63 mg/mL, 2.5 mg/mL, and 1.25 mg/mL, respectively, as shown in Table 12, while their bactericidal concentrations were all higher than 2.5 mg/mL. Amphotericin B exhibited inhibitory concentrations of 0.0004 mg/mL against *C. glabrata*, while for *C. albicans*, inhibition was observed at 0.063 mg/mL. These findings indicate that the fungal strains employed in this study are susceptible to the reference antifungal agent Amphotericin B.

## 3. Materials and Methods

All chemicals and reagents used in this study were of analytical grade. Ultrapure distilled water was produced using a Purite Selection Fusion system from Lasec^®^ Pty Ltd. (Cape Town, South Africa). Silver nitrate (AgNO_3_, 99.8%) was purchased from Rochelle Chemicals and Lab Equipment^®^ Pty Ltd. (Johannesburg, South Africa). *Dacryodes edulis* (G. Don.) H. J. Lam. stem bark was collected from Bertoua, East Region of Cameroon (specimen voucher number 65618/HNC). The papain-like protease (SARS-CoV-2) kit was purchased from Inqaba Biotech^®^ (Johannesburg, South Africa).

### 3.1. Plant Material Preparation and Plant Extraction

The stem bark was washed and chopped into cubed pieces, and then dried at room temperature under shadow for 20 days to remove moisture from the plant materials. The dried stem barks were ground using a Retch GmbH^®^ Cutting Mill SM 100 (North Rhine-Westphalia, Germany) into almost a fine powder. A maceration-type extraction was carried out by following a method by Wego Kamgaing et al. (2023) with slight modifications [29]. Briefly, 2 L of distilled water was added to 200 g of the stem bark powder and shaken for 24 h at ambient temperature. The solution was filtered using Sigma-Aldrich^®^ Whatman^®^ No.1 filter paper (Sigma-Aldrich, Waltham, MA, USA). Plant extraction was performed in triplicates. The combined filtrate from the three successive extractions was then freeze-dried using a benchtop SP Scientific^®^ VirTis Advantage Pro lyophilizer, Wizard (SP Industries, Inc., Warminster, PA, USA). A powdered *D. edulis* water extract sample (14.75 g) was obtained, which was stored at room temperature in an airtight container before further use.

### 3.2. Phytochemical Screenings of D. edulis Water Extract

Preliminary phytochemical screenings of the aqueous *D. edulis* water extract were conducted using standard procedures. These qualitative experiments aimed to determine the presence or absence of specific compound classes in the aqueous extract of *D. edulis*, based on previous phytochemical reports of the plant.

Terpenoids: To investigate the presence of terpenoids, 50 mg of the crude extract was mixed with 0.5 mL of chloroform. Concentrated sulfuric acid (H_2_SO_4_) of 0.3 mL was added to the mixture. The presence of terpenoids was confirmed by the formation of a brownish coloration at the interface of the mixture [119].

Saponins: For the presence of saponins, 50 mg of extract was diluted with distilled water and made up to 20 mL. The suspension was shaken in a graduated cylinder for 15 min. A two cm layer of foam indicated the presence [19,119].

Steroids: A powdered sample of 10 mg was dissolved in 0.1 mL of acetic acid separately; solutions were cooled, followed by the addition of a few drops of concentrated H_2_SO_4_. A color development from violet to blue or bluish-green was taken as a sign of the presence of a steroidal ring [29,119].

Anthraquinones: Crude extract of 10 mg was mixed with 0.1 mL of chloroform and shaken for 5 min. The solution was filtered, and then the filtrate was shaken with an equal volume of 10% ammonia solution. A pink-violet/red color in the ammoniacal layer (lower layer) indicated the presence of anthraquinone [29,119].

Tannins: A few drops of a 1% ferric chloride solution were added after mixing 100 mg of the powdered sample with 2 mL of distilled water and filtering. The appearance of blue-black, green, or blue-green precipitate forms indicated that tannins are present [19,29,119].

Phenols: A crude extract of 50 mg was mixed with 2 mL of a 5% ferric chloride solution. A blue coloration of the solution was taken as a confirmation of the presence of phenols [29,119].

### 3.3. High-Resolution Liquid Chromatography–Tandem Mass Spectrometry (HR-LC-MS/MS) Analysis of the D. edulis Water Extract

The sample was prepared into a 1 mg/mL solution using Merck Millipore^®^ ultra-pure water (Merck, Darmstadt, Germany) and filtered with a 0.45 μm Merck Millex^®^ nylon syringe filter (Darmstadt, Germany). For analysis, a high-resolution Waters^®^ Xevo G2 Quadrupole time-of-flight (QTOF) mass spectrometer (Waters, Manchester, UK) was used, coupled with a Waters^®^ Acquity ultra-performance liquid chromatograph (UPLC) (Waters, Milford, MA, USA). Data from the QTOFMS was analyzed using Chromalynx XS application manager software (Waters^®^ Corporation, Masslynx v 4.2).

### 3.4. Experimental Design and Optimization of Silver Nanoparticles

AgNPs synthesis was achieved by following a method previously described by Melkamu and Bitew (2021) with slight modifications [21]. Briefly, different concentrations of AgNO_3_ solution were added to the *D. edulis* water extract solution at a 1:1 ratio and varying temperatures while stirring at 1000 rpm. The formation of AgNPs was confirmed by the change in preparation color from pale yellow to dark brown to almost black color [21,120,121,122].

The formulation was then transferred to centrifuge tubes and left in a dark place while cooling for at least 1 h. The resulting AgNPs were centrifuged at 3500 rpm for 20 min. Afterwards, the pellets were washed with double-distilled water and centrifuged for another 20 min, and the process was repeated three times.

Every experiment was carried out in a randomized manner to minimize bias and ensure the reliability of the results. Randomization helps to account for uncontrolled variables or systematic errors that could influence the outcome. The optimization was carried out using a central composite design (CCD) developed using the design of experiments (DoE) as a quality-by-design (QbD) tool.

The selection of formulation and process parameters for the QbD study was guided by three considerations: prior literature on phyto-mediated AgNPs synthesis, the mechanistic roles of each variable in nanoparticle nucleation and growth, and preliminary laboratory trials. AgNO_3_ concentration was chosen as the silver precursor level governing ion availability and particle growth; *D. edulis* extract concentration was selected because it determines the amount of phytochemicals available for reduction and capping; reaction time was included to capture the kinetics of nanoparticle formation; and temperature was selected because it influences reduction rate, nucleation, particle growth, and surface stabilization. The factor ranges were defined from preliminary experiments as the boundaries within which nanoparticle formation occurred reproducibly without gross precipitation, excessive agglomeration, or visually unstable dispersions while still allowing for sufficient variation to detect effects on the selected CQAs.

In CCD, a quadratic model assists in determining the optimal reaction conditions in the optimization of chemical reactions that would maximize yield or minimize undesirable byproducts [21,86,123,124,125]. A summary of the CCD experiments is provided in Table 13.

All samples obtained from the CCD design experiments were synthesized and characterized and optimization was performed to obtain suitable surface plasmon resonance (SPR), zeta potential (ZP), average particle size (PS), and polydispersity index (PDI).

Statistical Analysis of Variance (ANOVA) was conducted using Design Expert^®^ statistical software v13.0.1.0 (Stat-Ease, Inc., Minneapolis, MN, USA) to analyze the main and combined effects of the considered parameters [126]. This analysis was utilized to develop empirical models to assess the impact of these factors on the particle size (PS), zeta potential (ZP), polydispersity index (PDI), and surface plasmon resonance (SPR).

### 3.5. Characterization of the AgNPs

The synthesized AgNPs were characterized for their optical properties, size distribution, surface functionalization, morphology, and crystallinity. A range of analytical techniques were used, namely, ultraviolet_–_visible (UV-Vis) spectroscopy, dynamic light scattering (DLS) wave analysis, Fourier-transform infrared (FTIR) spectroscopy, transmission electron microscopy (TEM), scanning electron microscopy (SEM), and powder X-ray diffraction (pXRD).

### 3.6. Ultraviolet-Visible Spectroscopy (UV-Vis) Analysis

The bio-reduction of silver nitrate (Ag^+^) to form AgNPs was monitored using UV-Vis spectroscopy. This technique involved tracking the intensity of the SPR within the visible range of 400–500 nm over time to determine the formation of AgNPs [110,127,128]. To characterize the AgNPs, an Agilent^®^ Cary 60 UV-Vis spectrophotometer (Agilent Technologies, Santa Clara, CA, USA) was used.

### 3.7. Particle Size and Zeta Potential

Dynamic light scattering (DLS) is used to determine the particle size and size distribution of nanoparticles (NPs) in solution. By analyzing light-scattering properties and Brownian motion, it calculates the average diameter of the nanoparticles [129]. Meanwhile, the surface ZP of AgNPs is measured to assess the potential stability of the colloidal nanosuspension [130]. PDI measures the uniformity of AgNPs within a sample [78]. In this work, the PS, ZP, and PDI were analyzed using a Microtrac MRB^®^ Nanotrac Wave II (Microtrac MRB, York, PA, USA).

### 3.8. Formulation Optimization

Optimization of the biosynthesized AgNPs using *D. edulis* water extract was done to develop a novel synthesis method for this drug-delivery system. This method would be cost-effective, eco-friendly and require less energy than conventional synthesis methods [2].

Numerical optimization was conducted to ascertain an optimal formulation composition and corresponding parameters that would guarantee the production of AgNPs with maximum stability, aimed at targeting infectious microbes. Various strategies are used to optimize process and formulation parameters; however, numerical optimization serves as a thorough and effective strategy for any ongoing optimization endeavor [131]. Numerical optimization identifies a spatial point that maximizes the desire function by altering the attributes of a target through the adjustment of its significance [131].

The synthesis of biosynthesized AgNPs using *D. edulis* water extract was optimized to develop a novel formula. The solution was chosen based on the highest percentage of desirability, as indicated by the software. The possible synthesis parameters are shown in Table 14.

### 3.9. Fourier-Transform Infrared Spectroscopy (FTIR)

FTIR analyses were performed to identify the functional groups of secondary metabolites in the *D. edulis* water extract, which may be responsible for the reduction, stabilization, and capping of AgNPs [21,96]. Approximately 1 mg of powdered AgNPs and freeze-dried *D. edulis* water extract was placed on the diamond ATR (attenuated total reflection) accessory window. FTIR spectra were obtained using an Agilent^®^ Cary 630 FTIR spectrophotometer (Santa Clara, CA, USA) in the 4000–400 cm^−1^ range with a spectral resolution of 2 cm^−1^ in transmittance mode. The background spectrum was collected using Agilent^®^ MicroLab PC Software, Version 5.6.2135.0 (Santa Clara, CA, USA).

### 3.10. Transmission Electron Microscopy (TEM)

TEM allows for precise measurement of particle size and distribution, as well as determining the shape of NPs. TEM analysis was used to examine the morphology, size, and dispersion of the NPs. The AgNPs were visualized on a Jeol^®^ JEM-1010 (JEOL Ltd., Tokyo, Japan) Field Emission Electron Microscope.

### 3.11. Scanning Electron Microscopy (SEM)/Energy-Dispersive X-Ray Scanning Electron Microscope (EDX)

SEM was used to examine surface morphology, while EDS was used to determine the elemental composition of the AgNPs [132]. Using a Quorum^®^ Q150T ES sputter coater (Quorum Technologies Ltd., Laughton, UK), the samples of the AgNPs were coated with carbon. The surface morphology of the AgNPs was visualized on a Carl Zeiss^®^ Supra 55VP Scanning Electron Microscope (Carl Zeiss, Oberkochen, Germany), coupled to an INCA Penta FETx3 Oxford Instruments^®^ EDS analyzer (Oxford Instruments, High Wycombe, UK) at magnifications of 10.000× to 15.000× [133].

### 3.12. Powder X-Ray Diffraction (pXRD)

*p*XRD was used to study the crystalline nature of the NPs as well as the measurement of average particle size [21,132,134]. The spectra data was collected within an angular range of 10° to 90° (2θ). The *p*XRD spectral data was obtained by using a Bruker^®^ D2 Phaser diffractometer (Bruker, Billerica, MA, USA) [133]. The average crystalline sizes of the AgNPs were calculated using Debye–Scherrer’s Equation (3).

(3) $$D=\frac{K\lambda }{\beta cos\theta }$$

### 3.13. Biological Assays

#### 3.13.1. Cell Viability Assay

Evaluating kidney toxicity is particularly important when screening potential drugs, as kidneys are vital for maintaining body homeostasis [135,136]. Human embryonic kidney (HEK-293) cells (American Type Culture Collection, Rockville, MD, USA), were used to investigate the potential nanotoxicity of AgNPs. HEK-293 cells were treated with *D. edulis* water extract and AgNPs solutions. Briefly, a methodology by Ghanbar et al. (2017) was followed with slight modifications [137]. The cells were treated with 10 µL of the respective samples (AgNPs and plant extract), seeded at a density of 2.5 × 10^4^ cells/well at 100 μL in a 96-well plate and incubated for 24 h at 37 °C in a humidified atmosphere containing 5% CO_2_. The metabolic activity of the cells was measured by assessing the reduction of MTT to formazan crystals. Absorbance was measured at 570 nm to evaluate cell viability. Cell viability was calculated using Equation (4).

(4) $$Cell\;viability\left(\%\right)=\left(\frac{Absorbance\;of\;treated\;sample}{Absorbance\;of\;negative\;control}\right)\times 100$$

#### 3.13.2. Papain-like Protease (PL-Pro) Enzyme Assay

The PL-pro enzyme kinetics assay kit (BPS Bioscience^®^, San Diego, CA, USA) was conducted as per the manufacturer’s instructions and as previously described by Węglarz-Tomczak et al. [51] to evaluate the inhibitory potential of AgNPs and *D. edulis* water extract (test samples). The test samples were added at 10 μL into separate wells of a 96-well microplate at concentrations of (1000 μg/mL to 50 μg/mL). Positive controls included dithiothreitol (DTT; 1 mM) inhibitor buffer and added at 10 µL into separate wells. Inhibitor controls included 5-amino-2-methyl-N-[(1R)-1-(1-naphthalenyl)ethyl]benzamide (GRL0617; 500 µM) prepared in DTT inhibitor buffer and added at 10 µL into separate wells. A solution of DTT inhibitor buffer was used as a negative control. A PL-pro enzyme solution (30 µL; 0.5 ng/µL) was added to each well and the reaction mixture was preincubated at 37 °C for 30 min to allow the enzyme to interact with the inhibitors or test samples (Table 15). The reaction was initiated by adding a chromogenic substrate solution (10 µL; 125 µM) to all the wells other than the blank and the plate further incubated at 37 °C for 60 min. Fluorescence was measured at an excitation of 355 nm and emission of 460 nm using a Spectra Max^®^ iD3 (Molecular Devices, San Jose, CA, USA) microplate reader with blank values subtracted from all other values.

#### 3.13.3. Neuraminidase (NA) Activity Assay

The NA-FluorTM Influenza Neuraminidase Assay Kit (Thermo Fisher Scientific^®^, Waltham, MA, USA) was used to test the effects of AgNPs and *D. edulis* water extract. The viral neuraminidase was tested on H1N1 strains, as per the manufacturer’s instructions and as previously described by Rajasekaran et al., 2013 [138]. The neuraminidase activity test kit uses Amplex Red™ (Molecular Devices, Eugene, OR, USA) to detect the presence of hydrogen peroxide (H_2_O_2_), which is generated by the oxidation of desialylated galactose from galactose oxidase. A working solution containing Amplex Red™ reagent (50 µL; 100 µM), horseradish peroxidase (HRP) (10 µL; 0.2 units/mL), galactose oxidase (100 µL; 4 units/mL), and fetuin (250 µL; 500 µg/mL) and 4.59 mL 1× reaction buffer was prepared and a volume of 50 µL was added to each well. The samples were added at 50 μL into separate wells of a 96-well microplate at concentrations of (1000 μg/mL to 50 μg/mL). Positive controls included a neuraminidase solution (1–5 U/mL), which was prepared by diluting the neuraminidase stock solution in the 1× reaction buffer. Additionally, a H_2_O_2_ positive control (10 µM) was prepared by diluting H_2_O_2_ working solution with the 1× reaction buffer; 50 μL of each control was added to separate control wells. A diluted neuraminidase solution was used as a negative control. The plate was initially incubated at 37 °C for 30 min, protected from light, allowing for continuous measurement of absorbance at multiple time points to monitor reaction kinetics. Absorbance was measured using a Spectra Max^®^ iD3 (CA, USA) microplate reader at 560 nm, with background correction performed by subtracting values from the negative control.

#### 3.13.4. Antimicrobial Assay

The antimicrobial activity of *D. edulis* water extract and AgNPs was evaluated using a microdilution assay against various bacterial and fungal strains (*Staphylococcus aureus* (ATCC 25923), *Streptococcus pyogenes* (ATCC 8665), *Escherichia coli* (ATCC 25922), *Pseudomonas aeruginosa* (ATCC 27853), *Candida albicans* (ATCC 90028), *Candida glabbrata* (ATCC 2850)) associated with nosocomial infections. The strains were obtained from the oral microbiology laboratory in the Department of Oral Biological Sciences, Faculty of Health Sciences, University of the Witwatersrand. These particular strains were chosen because they are commonly found in hospital environments and represent a substantial threat to individuals with weakened immune systems. Stock solutions of the compounds were prepared in Dimethyl sulfoxide (DMSO), and serial dilutions were performed in a 96-well microplate with bacterial suspensions (1 × 10^6^ CFU/mL). The plates were incubated for 24 h, followed by the addition of 0.04% p-iodonitrotetrazolium (INT) to assess bacterial growth, indicated by a color change. The minimal inhibitory concentration (MIC) was determined by the lowest concentration where no bacterial growth was observed. Ciprofloxacin (0.01 µg/mL) and Amphotericin B (0.01 µg/mL) were used as the positive controls for bacteria and fungi, respectively, and DMSO served as the negative control. The assay was performed in triplicate to ensure accuracy and reproducibility.

## 4. Conclusions

In this study AgNPs were successfully synthesized and optimized using an eco-friendly, green synthesis approach with *D. edulis* stem bark aqueous extract as a reducing, stabilizing, and capping agent. The optimized AgNPs demonstrated multi-targeted antiviral and antimicrobial activity, positioning them as promising candidates for addressing the rising threat of multidrug-resistant pathogens and viral infections, including SARS-CoV-2 and influenza H1N1. The DoE approach enabled precise optimization of synthesis parameters, ensuring reproducibility, stability, and enhanced bioactivity. Characterization using UV-Vis spectroscopy, FTIR, TEM, DLS, and pXRD confirmed the formation of stable, well-defined nanoparticles with desirable physicochemical properties for biomedical applications. Biological assays revealed superior antiviral, antibacterial, and antifungal activity of AgNPs compared to the crude plant extract, reinforcing the synergy between phytochemicals and silver nanoparticles as a potent biofunctional material.

This study integrated green nanotechnology and traditional medicine, leveraging the biological richness of *D. edulis* for scalable AgNP production with enhanced therapeutic potential. Unlike conventional chemical or physical AgNP synthesis methods, which often require toxic reagents, high-energy inputs, and complex purification steps, our approach ensures sustainability, cost effectiveness, and biocompatibility. Additionally, the discovery of multi-modal bioactivity—from SARS-CoV-2 PL-pro inhibition to neuraminidase inhibition and broad-spectrum antibacterial efficacy—highlights the vast biomedical potential of these nanoparticles in nanomedicine and targeted drug delivery systems. The ability to modulate physicochemical properties through QbD optimization further enhances the clinical translatability of these nanoparticles for infectious disease therapeutics.

This research requires exploration of several key areas for further advancement. Firstly, the predictive models for SPR and PDI yielded lower R^2^ values (<0.5), while the model for zeta potential showed a moderate correlation (R^2^ = 0.6405). Given that this study represents a seminal exploration of *D. edulis*-derived AgNPs, our DoE approach was intended to inform process optimization rather than serve as a broadly generalizable predictive model. Notably, all confirmation experiments fell within one standard deviation and 95% prediction intervals of the model residuals, indicating experimental alignment despite the lower statistical strength. As such, model revalidation was not pursued. Future endeavors will tackle this by enhancing replication at pivotal factor settings, fine-tuning factor ranges to more accurately reflect curvature effects and integrating weighted multi-response optimization that emphasizes essential functional parameters, such as zeta potential and PDI, in accordance with their significance in nanoparticle stability and application-specific efficacy. This will strengthen the optimization process and enhance the dependability of predictive modeling for green-synthesized metallic nanoparticles. Also, mechanistic studies on nanoparticle–virus interactions are essential to clarify the specific mechanisms by which AgNPs affect viral proteins, including their potential roles in viral entry, replication, and immune modulation. Molecular docking and in silico models may enhance these studies by forecasting the binding efficiency of AgNP to viral surface proteins or intracellular targets.

Furthermore, in vivo studies and toxicity profiling represent essential subsequent phases. The MTT assay conducted on HEK-293 cells demonstrated significant biocompatibility at therapeutic doses; however, further investigation through animal models and histopathological analysis is essential to evaluate pharmacokinetics, biodistribution, and long-term safety. This will facilitate the development of optimal dosing regimens and possible clinical applications.

The incorporation of AgNPs into secondary drug delivery systems, including hydrogels, nanofiber scaffolds, or microneedle patches, may facilitate temperospatial and prolonged antimicrobial release, thereby minimizing systemic toxicity and improving efficacy. The potential synergy between AgNPs and conventional antiviral or antibiotic therapies warrants investigation to address drug resistance and improve therapeutic outcomes.

Although the optimized AgNPs demonstrated favorable physicochemical characteristics and promising antiviral and antimicrobial activity, dedicated stability and release studies in physiologically relevant media remain necessary in future work to further support translational applicability.

This study facilitates a significant transformation in the integration of nanobiotechnology and traditional medicine, presenting a scalable, biocompatible, and multifunctional nanoplatform for the management of infectious diseases. The ongoing advancement of biofunctional AgNPs presents significant potential for future antimicrobial therapies, pandemic readiness, and innovations in global health.

## Figures and Tables

**Table 1:** Summary of the phytochemical screening of *D. edulis* water extract.

No	Phytochemical	Result
1	Terpenoids	++
2	Saponins	-
3	Steroids	-
4	Anthraquinones	+
5	Tannins	+
6	Phenols	++

Notes: ++ = highly present, + = present, - = not detected (number of repeats *n* = 3).

**Table 3:** Summary of experimental data for statistical models for responses monitored.

Response Factor	Response Surface Model					
	SD	R^2^ Value	Adj R^2^	Mean	Adeq Prec	C.V %
PS	90.40	0.8471	0.7043	222.40	7.6012	40.65
PDI	0.1358	0.2316	0.1087	0.2225	4.2353	61.01
ZP	25.57	0.6405	0.4513	−1.08	7.3920	23.60
SPR	28.47	0.4998	0.2365	451.93	5.0239	6.30

**Table 4:** ANOVA data for response surface quadratic model for particle size.

Source	Sum of Squares	df	Mean Square	F-Value	*p*-Value
Model	6.789 × 10^5^	14	48,496.15	5.93	0.0007
A-Conc AgNO_3_	2.026 × 10^5^	1	2.026 × 10^5^	24.79	0.0002
B-Conc *D. edulis*	656.64	1	656.64	0.0804	0.7807
C-Time	3653.15	1	3653.15	0.4470	0.5139
D-Temperature	2063.87	1	2063.87	0.2526	0.6226
AB	11,116.61	1	11,116.61	1.36	0.2617
AC	20,387.97	1	20,387.97	2.49	0.1351
AD	2245.65	1	2245.65	0.2748	0.6078
BC	6941.86	1	6941.86	0.8495	0.3713
BD	310.49	1	310.49	0.0380	0.8481
CD	1921.13	1	1921.13	0.2351	0.6348
A^2^	1.410 × 10^5^	1	1.410 × 10^5^	17.26	0.0008
B^2^	10,552.94	1	10,552.94	1.29	0.2736
C^2^	234.91	1	234.91	0.0287	0.8676
D^2^	2743.49	1	2743.49	0.3357	0.5709
Residual	1.226 × 10^5^	15	8171.94		

Note: df = degrees of freedom; significant factors reported in red.

**Table 5:** ANOVA data for response surface linear model for PDI.

Source	Sum of Squares	df	Mean Square	F-Value	*p*-Value
Model	0.1389	4	0.0347	1.88	0.1447
A-Conc. AgNO_3_	0.1023	1	0.1023	5.55	0.0266
B-Conc. *D. edulis*	0.0254	1	0.0254	1.38	0.2520
C-Time	0.0027	1	0.0027	0.15	0.7043
D-Temperature	0.0017	1	0.0017	0.09	0.7612
Residual	0.4609	25	0.0184		

Note: Significant values are reported in red.

**Table 6:** ANOVA data for response model for zeta potential.

Source	Sum of Squares	df	Mean Square	F-Value	*p*-Value
Model	22,131.75	10	2213.18	3.39	0.0107
A-Conc. AgNO_3_	2361.22	1	2361.22	3.61	0.0726
B-Conc. *D. edulis*	2508.96	1	2508.96	3.84	0.0649
C-Time	317.12	1	317.12	0.4851	0.4945
D-Temperature	4195.43	1	4195.43	6.42	0.0203
AB	2191.76	1	2191.76	3.35	0.0828
AC	297.99	1	297.99	0.4559	0.5077
AD	10,686.99	1	10,686.99	16.35	0.0007
BC	199.25	1	199.25	0.3048	0.5873
BD	1936.20	1	1936.20	2.96	0.1015
CD	854.36	1	854.36	1.31	0.2671
Residual	12,420.05	19	653.69		

Note: Significant factors are reported in red.

**Table 7:** The ANOVA data for the response surface quadratic model for surface plasmon resonance.

Source	Sum of Squares	df	Mean Square	F-Value	*p*-Value
Model	15,389.61	10	1538.96	1.90	0.1101
A-Conc. AgNO_3_	185.95	1	185.95	0.2294	0.6375
B-Conc. *D. edulis*	182.52	1	182.52	0.2251	0.6406
C-Time	424.29	1	424.29	0.5233	0.4782
D-Temperature	2979.99	1	2979.99	3.68	0.0704
AB	4887.02	1	4887.02	6.03	0.0239
AC	2272.50	1	2272.50	2.80	0.1105
AD	1.06	1	1.06	0.0013	0.9715
BC	9.56	1	9.56	0.0118	0.9147
BD	762.52	1	762.52	0.9405	0.3443
CD	2410.91	1	2410.91	2.97	0.1009
Residual	15,404.25	19	810.75		

Note: Significant values are reported in red.

**Table 8:** Optimized reaction synthesis parameters.

X_1_ mg/mL	X_2_ mg/mL	X_3_ min	X_4_ °C	PS nm	PDI	ZP mV	SPR nm	D
1.00	0.50	72.24	49.31	137	0.29	−30	430	0.96

**Table 9:** Summary of the results for the synthesized AgNPs using optimum formulation and process parameters.

Response	Predicted Value	Experimental Value	% PE	Model SD	Residual (Exp − Pred)	Standardized Residual	95% PI Lower	95% PI Upper
PS (nm)	137.00	156.00	−13.86	90.4000	19.00	0.21	−43.80	317.80
PDI	0.29	0.34	−17.24	0.1358	0.05	0.37	0.0184	0.5616
ZP (mV)	−30.00	−22.00	−26.67	25.5700	8.00	0.31	−81.14	21.14
SPR (nm)	430.00	420.00	2.33	28.4700	−10.00	−0.35	373.06	486.94

**Table 10:** The Papain-like protease enzyme assay (IC_50_ values from the enzyme kinetics study).

Sample	IC_50_ Mean ± SD (µg/mL)
AgNPs	271.0 ± 15.00 ^b^
H_2_O extract of *D. edulis*	337.0 ± 23.00 ^c^
GRL0617	0.487 *^a^

IC_50_ results are presented as mean values ± standard deviation; alphabetical letters indicate *p* ˂ 0.05. * IC_50_ for GRL0617 presented according to the manufacturer instructions.

**Table 11:** IC_50_ values from the enzyme kinetics study of neuraminidase viability assay of optimized AgNPs and *D. edulis* water extract on H1N1.

Sample	IC_50_ Mean ± SD (µg/mL)
AgNPs	18.40 ± 0.04 ^b^
H_2_O extract of *D. edulis*	514.39 ± 86.37 ^c^
Oseltamivir	0.1769 ± 0.04 ^a^

IC_50_ results are presented as mean values ± standard deviation; alphabetical letters indicate *p* < 0.05.

**Table 12:** The antimicrobial activity assay of the AgNPs and *D. edulis* water extract using in mg/mL.

Samples	Gram Negative				Gram Positive				Fungi			
	*E. coli*		*P. aeruginosa*		*S. aureus*		*S. pyrogens*		*C. albicans*		*C. glabrata*	
	MIC	MBC	MIC	MBC	MIC	MBC	MIC	MBC	MIC	MFC	MIC	MFC
AgNPs	0.063	0.125	0.31	0.63	0.125	0.25	0.08	0.31	0.31	0.31	0.63	0.63
*D. edulis*	0.63	2.5	0.63	2.5	1.25	2.5	0.63	2.5	0.63	1.25	1.25	2.5

Key: *Escherichia coli* (*E. coli*), *Pseudomonas aeruginosa* (*P. aeruginosa*), *Staphylococcus aureus* (*S. aureus*), *Streptococcus pyogenes* (*S. pyogenes*), *Candida albicans* (*C. albicans*), and *Candida glabrata* (*C. glabrata*).

**Table 13:** The CCD experiment parameters for the synthesis of AgNPs.

Run	Conc. AgNO_3_ mM	Conc. *D. edulis* mg/mL	Time min	Temp °C
1	3	0.5	55	60
2	1	1	30	80
3	1	1	30	40
4	5	1	80	40
5	1	1.5	60	80
6	3	0.5	5	60
7	5	1.5	80	80
8	5	1.5	80	40
9	3	1.5	55	60
10	5	0.5	55	60
11	1	1	80	80
12	1	1.5	30	80
13	5	1	30	80
14	3	1.5	55	60
15	1	1.5	55	60
16	1	0.5	55	60
17	3	0.5	55	60
18	3	0.5	55	60
19	1	1	80	40
20	3	0.5	105	60
21	1	1.5	80	40
22	1	1.5	30	40
23	3	1.5	55	60
24	5	1	80	80
25	5	1.5	30	40
26	3	0.5	55	100
27	5	1.5	30	80
28	5	1	30	40
29	3	0.5	55	60
30	3	0.5	55	20

**Table 14:** Indicates the solutions for the possible synthesis of the optimized formulation constraints and optimized formulation conditions for the manufacture of optimized AgNPs.

Name		Goal	Lower Limit	Upper Limit
A: Time		Minimize	20 min	80 min
B: Temp		Minimize	20 °C	80 °C
C: Conc. AgNO_3_		Minimize	1 mg/mL	5 mg/mL
D: Conc. *D. edulis*		Minimize	0.5 mg/mL	1.5 mg/mL
SPR (nm)		In range	420 nm	496 nm
ZP (mV)		Minimize	−27 mV	38 mV
PDI		Minimize	0.058	0.6
PS (nm)		Minimize	85 nm	454 nm
No.	Conc. AgNO_3_ mg/mL	Conc. *D. edulis* mg/mL	Time min	Temp °C
1.	1.00	0.50	72.24	49.31

**Table 15:** Test components of the enzyme kinetics assay.

Positive Control	Inhibitor Control	Blank	Test Sample Solution
1. PL-pro enzyme (30 μL)	1. PL-pro enzyme (30 μL)	1. PL-pro enzyme (30 μL)	1. PL-pro enzyme (30 μL)
2. DTT Inhibitor buffer (10 μL)	2. GRL0617 (10 μL)	2. DTT inhibitor buffer (10 μL)	2. Test sample (10 μL)
3. Substrate solution (10 μL)	3. Substrate solution (10 μL)		3. Substrate solution (10 μL)

## Data Availability

Data will be made available on request.

## Supplementary Materials

The following supporting information can be downloaded at: https://www.mdpi.com/article/10.3390/molecules31111821/s1, Figure S1: BPI chromatogram of the water extract of *D. edulis*; Figure S2. MS/MS fragmentation patterns of identified compounds from the water extract of *D. edulis*.
