# Supplementary material for: Green Synthesis and Quality-by-Design Optimization of Dacryodes edulis-Derived Silver Nanoparticles with Broad-Spectrum Antiviral and Antimicrobial Activity
Source: Molecules. 2026 May 25;31(11):1821. doi: 10.3390/molecules31111821 (PMC13258639; doi:10.3390/molecules31111821)
Supplement: Supplementary file 1 [file molecules-31-01821-s001.zip › molecules-4203624-supplementary.pdf]

Green Synthesis and Quality-by-Design Optimization of *Dacryodes edulis*-Derived Silver Nanoparticles with Broad-Spectrum Antiviral and Antimicrobial Activity

Jabulile H. Xulu [1], Vuyelwa J. Tembu [2], Sharon Moeno [3], Bienvenu Tsakem [1], Vuyisile S. Thibane [4], Bwalya A. Witika [1,]* and Xavier Siwe Noundou [1,]*

[1] Department of Pharmaceutical Sciences, School of Pharmacy, Sefako Makgatho Health Sciences University, Pretoria 0204, South Africa; jabuhappinessxulu@gmail.com (J.H.X.); btsakem23@gmail.com (B.T.)
[2] Department of Chemistry, Faculty of Science, Tshwane University of Technology, Private Bag X680,
Pretoria 0001, South Africa; tembuvj@tut.ac.za
[3] Department of Oral Biological Sciences, School of Oral Health Sciences, Faculty of Health Sciences,
University of the Witwatersrand, Johannesburg 2193, South Africa; sharon.moeno@wits.ac.za
[4] Department of Biochemistry and Biotechnology, School of Science and Technology, Sefako Makgatho Health Sciences University, Pretoria 0204, South Africa; vuyisile.thibane@smu.ac.za
* Correspondence: bwalya.witika@smu.ac.za (B.A.W.); xavier.siwenoundou@smu.ac.za (X.S.N.)

Supplementary Material

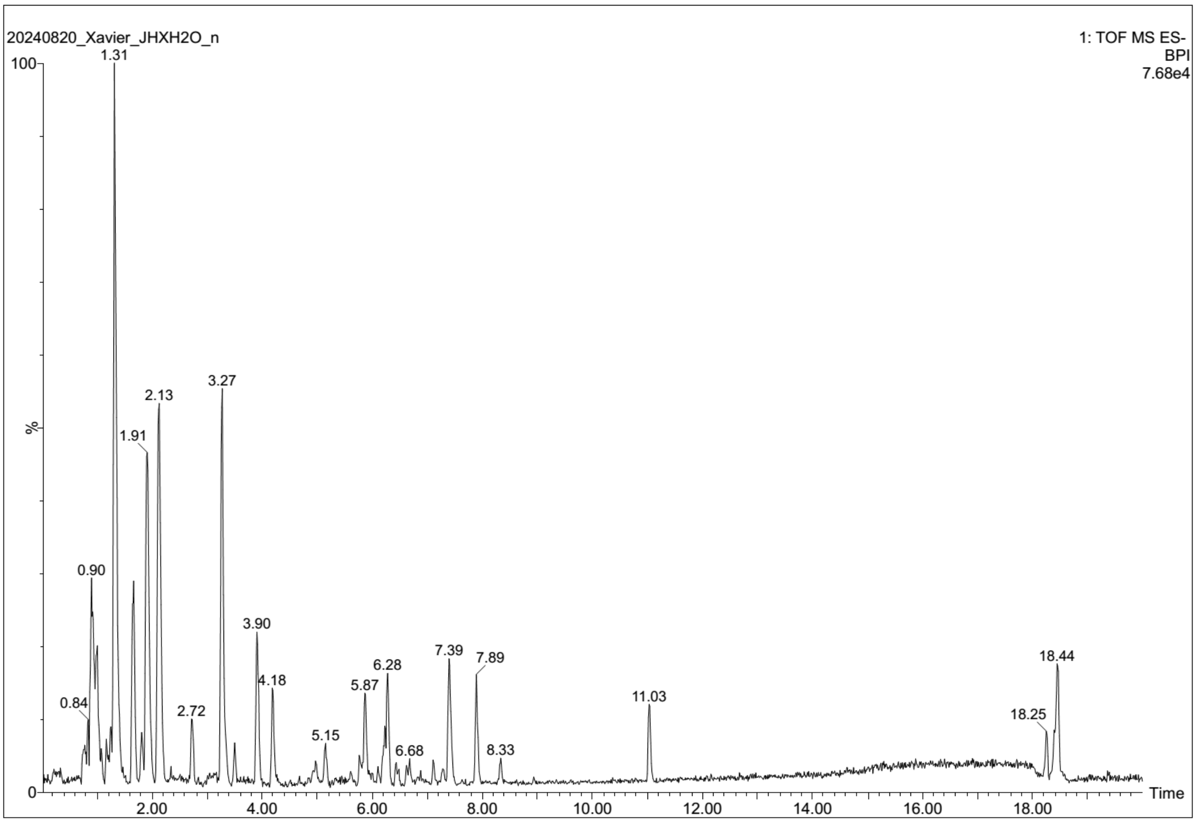

Figure S1. BPI chromatogram of the water extract of *D. edulis*

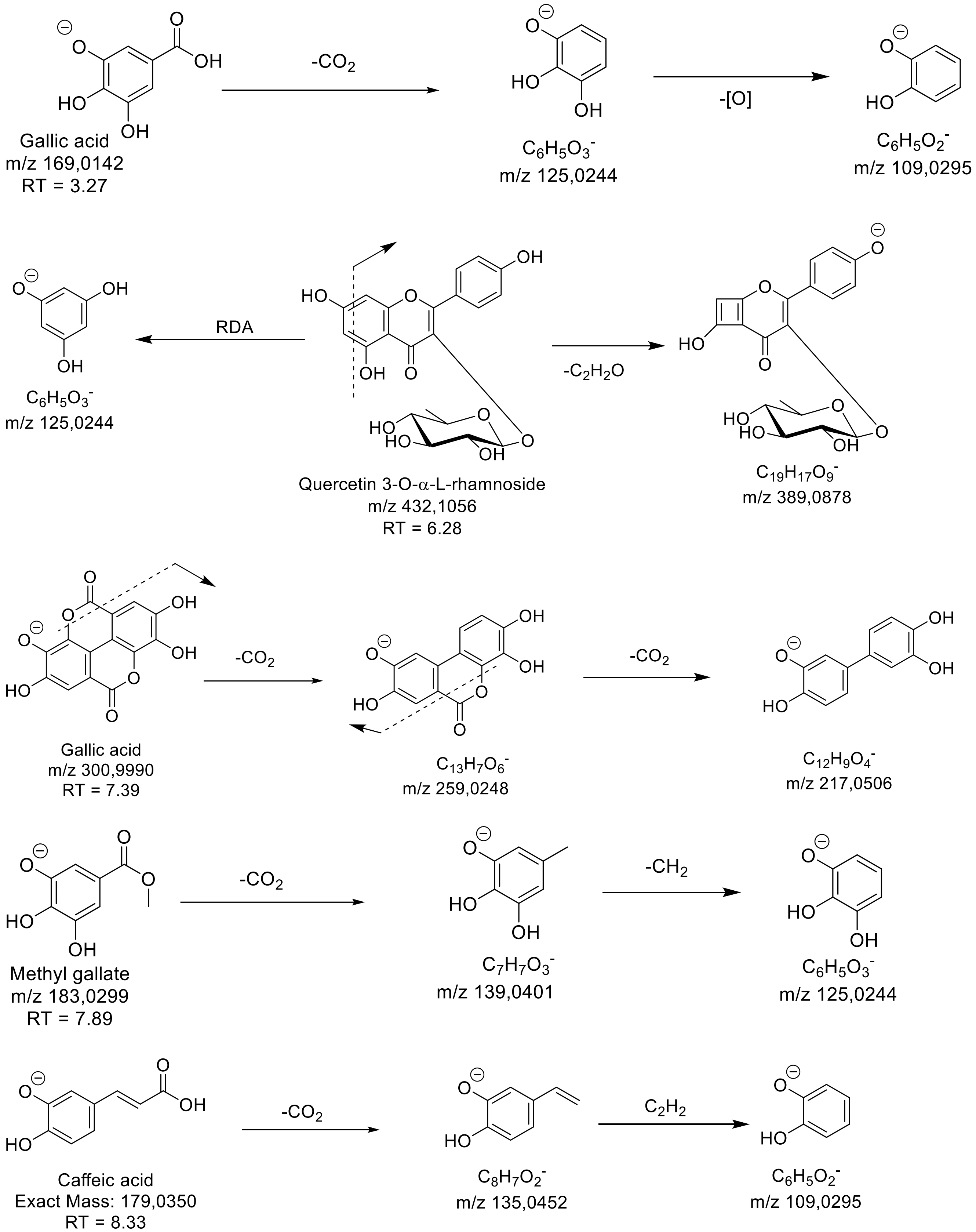

-CO2
-[O]
Gallic acid
m/z 169,0142
RT = 3.27
C6H5O3-
m/z 125,0244
C6H5O2-
m/z 109,0295
RDA
-C2H2O
C6H5O3-
m/z 125,0244
Quercetin 3-O-α-L-rhamnoside
m/z 432,1056
RT = 6.28
C19H17O9-
m/z 389,0878
-CO2
-CO2
Gallic acid
m/z 300,9990
RT = 7.39
C13H7O6-
m/z 259,0248
C12H9O4-
m/z 217,0506
-CO2
-CH2
Methyl gallate
m/z 183,0299
RT = 7.89
C7H7O3-
m/z 139,0401
C6H5O3-
m/z 125,0244
-CO2
C2H2
Caffeic acid
Exact Mass: 179,0350
RT = 8.33
C8H7O2-
m/z 135,0452
C6H5O2-
m/z 109,0295

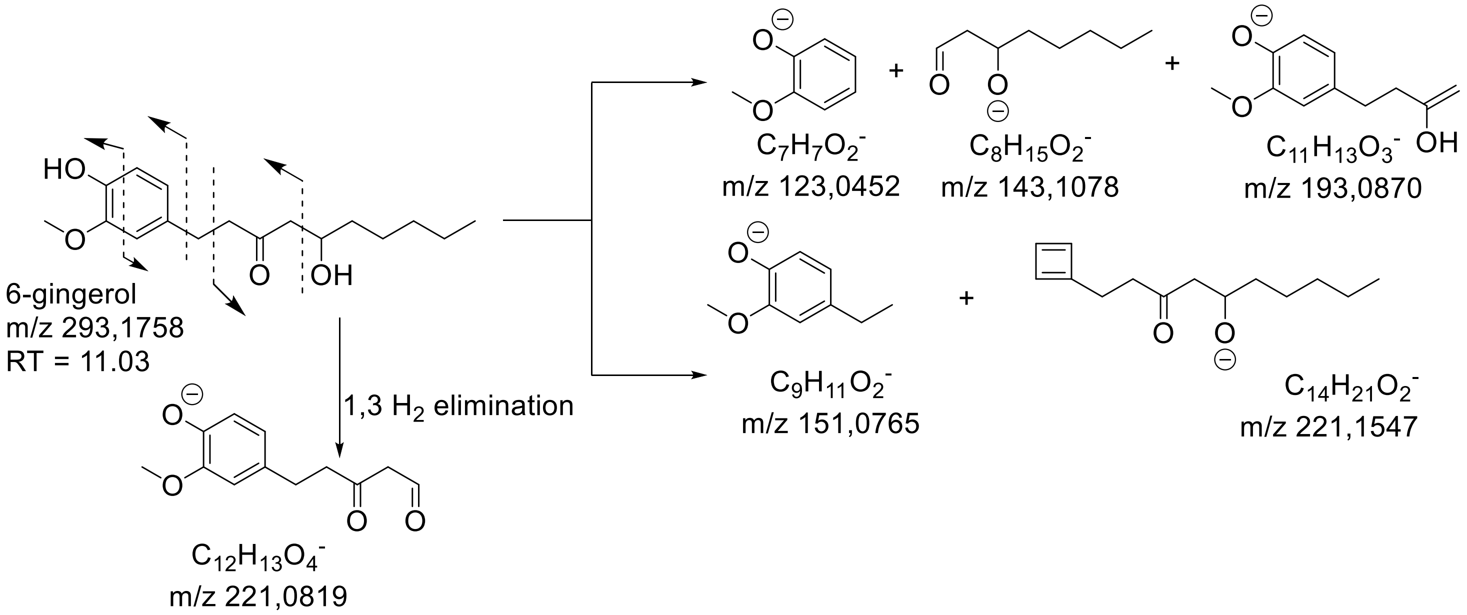

Figure S2. MS/MS fragmentation patterns of identified compounds from the water extract of D. edulis
